# Pregnancy Outcomes After Frozen-Thawed Embryo Transfer in the Absence of a Corpus Luteum

**DOI:** 10.3389/fmed.2021.727753

**Published:** 2021-09-10

**Authors:** Freya Waschkies, Luka Kroning, Thilo Schill, Arvind Chandra, Cordula Schippert, Dagmar Töpfer, Yvonne Ziert, Frauke von Versen-Höynck

**Affiliations:** ^1^Department of Obstetrics and Gynecology, Hannover Medical School, Hannover, Germany; ^2^Fertility Center Langenhagen, Langenhagen, Germany; ^3^Center for Reproductive Medicine, Bad Münder, Germany; ^4^Institute of Biostatistics, Hannover Medical School, Hannover, Germany

**Keywords:** birthweight, hypertensive disorders of pregnancy, pregnancy outcome, stimulated cycle, natural cycle, programmed cycle, corpus luteum, frozen-thawed embryo transfer

## Abstract

**Background:** Nowadays, frozen-thawed embryo transfer (FET) cycles represent a high proportion of fertility treatments worldwide. Recent studies suggest differences in pregnancy outcomes depending on the FET treatment protocol used. The reason for this is still unclear, but the number of corpora lutea (CL) at conception is discussed as a possible factor. This study aims to investigate whether maternal and neonatal outcomes for pregnancies following FET lacking a CL differ from FET with one or more CL in order to explore a potential link between CL absence and adverse pregnancy outcomes.

**Methods:** The study was designed as a retrospective, multi-center observational study with two cohorts after singleton live birth [0 CL cohort (FET in a programmed cycle, *n* = 114) and ≥ 1 CL cohort (FET in a natural or stimulated cycle, *n* = 68)]. Participants completed a questionnaire on the outcome of pregnancy and birth records were analyzed in a descriptive way. Multivariable logistic and linear regressions were performed in order to explore associations between CL absence and pregnancy outcomes. The strength of the agreement between the information in the survey and the diagnoses extracted from the files was assessed by Cohen's Kappa.

**Results:** The risk of hypertensive disorders of pregnancy was higher after FET in the absence of a CL compared to FET with CL presence (aOR 5.56, 95% CI 1.12 – 27.72). Birthweights and birthweight percentiles were significantly higher in the 0 CL group. CL absence was a predictor of higher birthweight (adjusted coefficient B 179.74, 95% CI 13.03 – 346.44) and higher birthweight percentiles (adjusted coefficient B 10.23, 95%, 95% CI 2.28 – 18.40) particularly in female newborns of the 0 CL cohort. While the strength of the agreement between the reported information in the survey and the actual diagnoses extracted from the files was good for the majority of outcomes of interest it was fair in terms of hypertension (κ = 0.38).

**Conclusion:** This study supports observations suggesting a potential link between a lack of CL at conception and adverse maternal and neonatal outcomes. Further investigations on causes and pathophysiological relationships are yet to be conducted.

## Introduction

Over the last two decades, the number of recorded assisted reproductive technology (ART) treatments has been continuously rising, with a 5.3-fold increase in Europe and a 4.6-fold increase in the USA from 1997 to 2016 (1).

Amongst ART treatments, frozen embryo transfer (FET) plays an increasingly central role (2) with delivery rates per embryo transfer in thawing cycles exceeding that in fresh cycles in the USA, Australia and New Zealand for years now (1). Commonly used cycle protocols in order to prepare the endometrium for FET include natural cycles (pure or modified), stimulated cycles (with ovulation induction), and programmed cycles (with hormonal replacement), of which the first two options imply corpus luteum (CL) presence—in opposition to the latter with CL absence (2). Of these three options FET in a programmed cycle is the most frequently applied protocol (3), with a current 75% share in Germany for example.

The increasingly important role of FET makes it imperative to ask which cycle protocol is most beneficial and safest in terms of both maternal and neonatal outcomes. Several studies suggest higher rates of adverse maternal outcomes, e.g., hypertensive disorders of pregnancy (HDP) (4–10), preeclampsia with preterm delivery (10), postpartum hemorrhage (11) and adverse neonatal outcomes, e.g., large for gestational age (LGA) birthweight (5, 8, 12–14), postterm birth, macrosomia (8, 12), and perinatal mortality (14), in pregnancies arising from FET compared to fresh embryo transfers and spontaneous conceptions.

However, the reasons behind this are still unknown. Recent studies imply a link between CL absence and specific maternal and neonatal outcomes, such as macrosomia (15), postterm birth (16), HDP (2), postpartum hemorrhage (15–17), and Cesarean section rate (15, 16, 18–20).

In programmed cycles, estradiol and progesterone are being substituted, which does not apply to other vasoactive products normally produced by the CL, like relaxin and vascular endothelial growth factor (21, 22). Recent findings indicate that those play a crucial role in the physiologic preparation for and adaptation to pregnancy, for instance maternal cardiovascular and renal changes in early pregnancy (23–26), regulation of endometrial function (23), preparation for implantation (27), and initial placentation (21). Malfunctions or absence of these processes have been linked to higher risks for developing adverse pregnancy outcomes like preeclampsia (23, 25, 27, 28).

Further investigations on the causal relations between CL number at conception might eventually have an impact on commonly used ART protocols, potentially leading to lower risks for adverse maternal and neonatal outcomes. Hence, we conducted a retrospective study aiming to assess maternal and neonatal outcomes after FET in a multi-center cohort in order to explore a potential link between CL absence and adverse pregnancy outcomes.

## Materials and Methods

### Participants

This retrospective study included information from all FET cycles conducted between June 1997 and November 2019 at three German Fertility Centers. Women with known singleton live birth from autologous oocytes after FET were initially contacted *via* telephone or alternatively *via* email between January and October 2019. After providing informed consent, participants agreed to the release of medical records by the delivery hospitals and completed a questionnaire. The survey included 16 questions regarding delivery hospital, maternal characteristics, the fertility treatment, the pregnancy of interest and previous pregnancies. The survey data acted as an indispensable presupposition for the request of medical records from the respective delivery hospitals. This procedure also enabled a comparison between survey answers and information from medical records as well as the assignment between participant and delivery hospital. A reminder was being sent after 4 weeks in case of non-response. Participation in the survey was online using the software SoSci Survey (versions 3.1.04 and 3.2.00) or if requested *via* mail. Participants were grouped according to the absence or presence of CL: those after FET in a programmed cycle (with hormonal replacement) were assigned to the 0 CL cohort and those after FET in a natural cycle (pure or modified) or FET in a stimulated cycle (ovulation induction using low-dose gonadotropins) were assigned to the ≥1 CL cohort.

### Endometrial Preparation Protocols

The choice of endometrial preparation protocol depended on physician or center preference and patient's characteristics. Endometrial preparation for the programmed cycle protocol included a vaginal ultrasound on day 2 or 3 of the menstrual cycle and the administration of 4–6 mg oral estradiol valerate per day. The dose was increased by 2–4 mg per day if the endometrial thickness didn't reach ≥7 mm on day 10–12 of the menstrual cycle. Once endometrial thickness reached ≥7 mm endometrial transformation was induced by 600 mg vaginal progesterone daily. Oral estradiol valerate and vaginal progesterone were continued until 10–12 weeks' gestation. In women using a natural cycle regimen the development of a dominant follicle was monitored by vaginal ultrasound and serum estradiol and luteinizing hormone (LH) analysis until ovulation was triggered by a subcutaneous human choriogonadotropin (hCG) injection in all but one participant. Vaginal progesterone for luteal phase support was administered at a dose of 200–300 mg daily. The endometrial preparation in a stimulated cycle started after a vaginal ultrasound with a daily injection of follicle stimulating hormone (37.5–75 IU) on day 3–5 of the menstrual cycle for ovulation induction. The dose was adjusted depending on BMI, previous ovarian response and ovarian reserve. Follicular growth was monitored by vaginal ultrasound, serum estradiol and LH analysis. Subcutaneous hCG was administered once the dominant follicle reached ≥17 mm. Luteal phase support in stimulated cycles corresponded to that for (modified) natural cycles and was continued until 10–12 weeks' gestation.

### Outcomes

Information on demographic characteristics and fertility treatment was extracted from the respective medical records of the fertility clinics, specifics on delivery, peripartal, and perinatal course from medical records requested from the hospital in which the participants delivered their children.

Primary maternal outcome was the incidence of hypertensive disorders of pregnancy under consideration of the current national (29) and international definitions (30), including gestational hypertension, preeclampsia, eclampsia, or HELLP-syndrome. Adjudication of a diagnosis was performed blinded, e.g., excluding information about the relevant study group, by a trained obstetrician. Secondary maternal outcomes were the incidences of postpartum hemorrhage, placenta-associated complications (placenta accreta, retained placenta, or placenta praevia), gestational diabetes and Cesarean section rate.

Neonatal outcomes were preterm birth (birth <37 gestational weeks), low (<2,500 g) and very low birthweight (<1,500 g), macrosomia (>4,000 g), small for gestational age (SGA) (<10th percentile), LGA (>90th percentile), neonatal morbidity (e.g., icterus, infection, sepsis, infant respiratory distress syndrome, and hypoglycemia), admission to intermediate care unit (ICU) or neonatal intensive care unit (NICU) and major birth defects.

### Statistical Analysis

Data distribution was examined using the Shapiro-Wilk test. Descriptive statistics are presented by number (*n*) and percentages for categorical variables and by mean and standard deviation or median and interquartile range for continuous variables.

Student's *t*-test or Mann-Whitney test were performed to identify the significant differences between mean values of two continuous variables. Chi-square (χ^2^) or Fisher's exact test were performed to test for significant differences in proportions of categorical variables between the two groups.

Multivariable logistic regression and multivariable linear regression analyses were conducted to calculate crude (OR) and adjusted odds ratios (aOR) as well as coefficient B with 95% confidence interval (CI) for maternal and neonatal outcomes. Adjustments were made for the effect of the following confounders which are well-known risk factors for the studied outcomes: age > 40 years, nulliparity, polycystic ovary syndrome (PCOS) or anovulation, intracytoplasmic sperm injection (ICSI), transfer of > 1 embryo (for maternal outcomes) and child's sex, nulliparity, HDP, placenta-associated complications, smoking during pregnancy, and gestational diabetes (for neonatal outcomes).

For the comparison between survey answers and verified diagnoses, we report the difference in prevalence. Cohen's Kappa function was used to calculate agreement between survey answers and verified diagnoses.

Inferential statistics are used in a descriptive manner. According to Fisher's approach *p*-values are interpreted as a metric measure of evidence against the respective null hypothesis. Thus, no global significance level was determined, and no adjustment for multiplicity was applied. However, *p*-values < 0.05 were considered statistically noticeable. All analyses were performed using the software SPSS, versions 26 and 27.

## Results

### Participants

In all three participating fertility clinics, overall 8,700 FET's were conducted between 1997 and 2019. This led to a total of 1,131 documented live births. Out of this group, 260 women were excluded because of multiple births and 24 because of lacking contact data. The cohort of contacted people comprised a number of 847, from which 612 were excluded due to unsuccessful contact, refusion of participation or no respond to the survey. Among the 235 completed surveys and informed consents, partially including information on various pregnancies, 238 eligible pregnancies resulted. In 56 cases we could not get access to the participants' medical records. The final number of singleton live births after FET with autologous oocytes included in the study was 182 of which 114 (62.6%) were performed in the absence of a CL (programmed FET cycles) and 68 in the presence of a CL [22 (12.1%) in natural FET cycles and 46 (25.3%) in stimulated FET cycles]. These data are outlined in a study flow diagram (Supplementary Figure 1).

Categorized by the fertility clinics, 43 (23.6%) of the participants were treated at center 1, 107 (58.8%) at center 2, and 32 (17.6%) at center 3. Participants' response rates to the survey were significantly divergent between the three fertility clinics—with a 82.1% response rate from patients who received their embryo transfer in center 1, 69.4% in center 3, and 60.3% in center 2 (*p* = 0.006).

### Baseline and Medical History Characteristics

Table 1 sets out baseline and medical history characteristics of the participants. Characteristics e.g., age, body-mass index (BMI), parity, participants' and partners' ethnicities, smoking and alcohol consumption during pregnancy, history of HDP in prior pregnancies and pre-existing comorbidities were comparable between both groups. In the 0 CL cohort, male infertility occurred more often while in the ≥1 CL group more participants had experienced recurrent pregnancy loss or more than one embryo had been transferred.

**Table 1:** Baseline and medical history characteristics^a^.

	**0 CL** **(*n* = 114)**	**≥1 CL** **(*n* = 68)**	***P*-value**
**Age**			
Maternal age at children's birth—years	35.0 ± 3.8	35.5 ± 3.7	0.38
Maternal age 18–29 years	8 (7.0)	4 (5.9)	>0.9
Maternal age 30–34 years	44 (38.6)	24 (35.3)	0.66
Maternal age 35–39 years	46 (40.4)	29 (42.6)	0.76
Maternal age ≥40 years	16 (14.0)	11 (16.2)	0.69
**BMI**			
Maternal BMI pre-pregnancy—kg/m^2^	23.1 ± 6.7	23.6 ± 5.2	0.76
Maternal BMI (kg/m^2^) pre-pregnancy <18.5	5 (4.5)	2 (3.0)	0.71
Maternal BMI (kg/m^2^) pre-pregnancy 18.5−24.9	69 (59.6)	42 (61.2)	0.89
Maternal BMI (kg/m^2^) pre-pregnancy 25−29.9	25 (22.4)	15 (22.3)	0.99
Maternal BMI (kg/m^2^) pre-pregnancy ≥30	13 (11.7)	8 (12.0)	0.95
Unknown	2 (1.8)	1 (1.5)	>0.9
**Parity**			
Parity <1	72 (63.2)	39 (57.4)	0.46
Parity ≥ 1	41 (35.9)	28 (41.1)	0.46
Unknown	1 (0.9)	1 (1.5)	>0.9
**Participant ethnicity**			
European	108 (94.7)	68 (100)	0.09
Asian	4 (3.5)	0 (0.0)	0.30
African	1 (0.9)	0 (0.0)	>0.9
Other	1 (0.9)	0 (0.0)	>0.9
**Partner ethnicity**			
European	111 (97.4)	68 (100)	0.29
Asian	2 (1.8)	0 (0.0)	0.53
African	0 (0.0)	0 (0.0)	NA
Other	1 (0.9)	0 (0.0)	>0.9
**Cause of infertility^b^**			
Male factor	105 (92.1)	50 (73.5)	0.002
Tubal	14 (12.3)	12 (17.6)	0.28
Uterine	18 (15.8)	13 (19.1)	0.50
Ovarian	24 (21.1)	7 (10.3)	0.07
PCOS or anovulation	23 (20.2)	17 (25.0)	0.39
Endometriosis	11 (9.6)	11 (16.2)	0.17
Recurrent pregnancy loss	7 (6.1)	12 (17.6)	0.01
Other	32 (28.1)	18 (26.5)	0.91
Unknown	2 (1.8)	2 (2.9)	0.63
**Fertility treatment**			
Sperm source partner	113 (99.1)	66 (97.1)	0.29
Sperm source donor	1 (0.9)	2 (2.9)	0.55
Number of oocytes retrieved	14.8 ± 6.4	16.0 ± 9.3	0.97
**ICSI**			
ICSI treatment	93 (81.6)	49 (72.1)	0.32
Unknown	3 (2.6)	4 (5.9)	0.43
**Embryo transfer**			
Embryos transferred > 1	87 (76.3)	61 (89.7)	0.02
Unknown	3 (2.6)	2 (2.9)	>0.9
**Smoking and alcohol during pregnancy**			
Smoking during pregnancy	3 (2.6)	1 (1.5)	>0.9
Alcohol consumption during pregnancy	0 (0.0)	0 (0.0)	NA
**History of HDP in prior pregnancy**			
Preeclampsia or HELLP-syndrome in prior pregnancy	2 (1.8)	1 (1.5)	>0.9
Unknown	13 (11.4)	3 (4.4)	0.17
**Pre-existing diseases**			
Autoimmune disease	11 (9.6)	8 (11.8)	0.73
Chronic hypertension	4 (3.5)	3 (4.4)	> 0.9
Cardiovascular disease	2 (1.8)	0 (0.0)	0.52
Chronic kidney disease	2 (1.8)	1 (1.5)	>0.9
Thrombophilia	5 (4.4)	8 (11.8)	0.08
Diabetes Type 1 or 2	3 (2.6)	3 (4.4)	0.68
Hypothyroidism	42 (36.8)	18 (26.5)	0.10
Unknown	5 (4.4)	0 (0.0)	0.16

a *Data are presented as mean ± standard deviation, median ± interquartile range or as number (% of total)*.

b *Indications may overlap, does not sum up to 100%*.

*BMI, body-mass index; CL, corpus luteum; HDP, hypertensive disorder of pregnancy; ICSI, intracytoplasmic sperm injection; NA, not applicable; PCOS, polycystic ovary syndrome*.

### Maternal Outcomes

After adjustment for potential confounders, FET in the absence of a CL was associated with a higher risk of HDP (aOR 5.56, 95% CI, 1.12 – 27.72) when compared to FET with a CL. There was no difference in the incidences of postpartum hemorrhage, placenta-associated complications, gestational diabetes and Cesarean section rate (Table 2) or in incidences of preeclampsia subtypes with regards to onset and severity of disease (Table 3).

**Table 2:** Maternal outcomes of singleton birth after frozen-thawed embryo transfer in the absence (0 CL) or presence (≥1 CL) of a corpus luteum^a^.

**Outcomes**	**0 CL**	**≥1 CL**	***P*-value**	**Crude OR (95% CI)**	***P*-value**	**Adjusted OR^**d**^ (95% CI)**	***P*-value**
	**(*n* = 114)**	**(*n* = 68)**				
Hypertensive disorders of pregnancy^b^	15 (13.2)	3 (4.4)	0.06	3.28 (0.91 – 11.79)	0.07	5.56 (1.12 – 27.72)	0.04
Postpartum hemorrhage	7 (6.1)	2 (2.9)	0.49	2.16 (0.44 – 10.71)	0.35	2.05 (0.40 – 10.42)	0.39
Placenta-associated complication^c^	11 (9.6)	3 (4.4)	0.20	2.31 (0.62 – 8.61)	0.21	3.41 (0.72 – 16.26)	0.12
Gestational diabetes	16 (14.0)	6 (8.8)	0.30	1.69 (0.63 – 4.54)	0.30	1.94 (0.66 – 5.74)	0.23
Cesarean section	55 (48.3)	25 (36.8)	0.13	1.60 (0.87 – 2.97)	0.13	1.72 (0.87 – 3.43)	0.12

a *Data are presented as number (% of total)*.

b *Hypertensive disorders of pregnancy includes one of the following: gestational hypertension, preeclampsia, preeclampsia with severe features, HELLP-syndrome*.

c *Placenta-associated complication includes at least one of the following: placenta praevia, retained placenta, placenta accreta*.

d *Adjustments were made for the following confounders: maternal age > 40 years, nulliparity, PCOS or anovulation, ICSI treatment and transfer of > 1 embryo*.

*CL, corpus luteum; OR, odds ratio*.

**Table 3:** Preeclampsia subtypes in singleton birth after frozen-thawed embryo transfer in the absence (0 CL) or presence (≥1 CL) of a corpus luteum^a^.

**Preeclampsia subtypes**	**0 CL (*n* = 114)**	**≥1 CL (*n* = 68)**	***P*-value**
Early-onset (<34 weeks)	2 (1.8)	0 (0.0)	0.53
Late-onset (> 34 weeks)	7 (6.1)	3 (4.4)	0.75
Severe features	5 (4.4)	1 (1.5)	0.41

a *Data are presented as number (% of total)*.

*CL, corpus luteum*.

### Neonatal Outcomes

**Figure 1:**
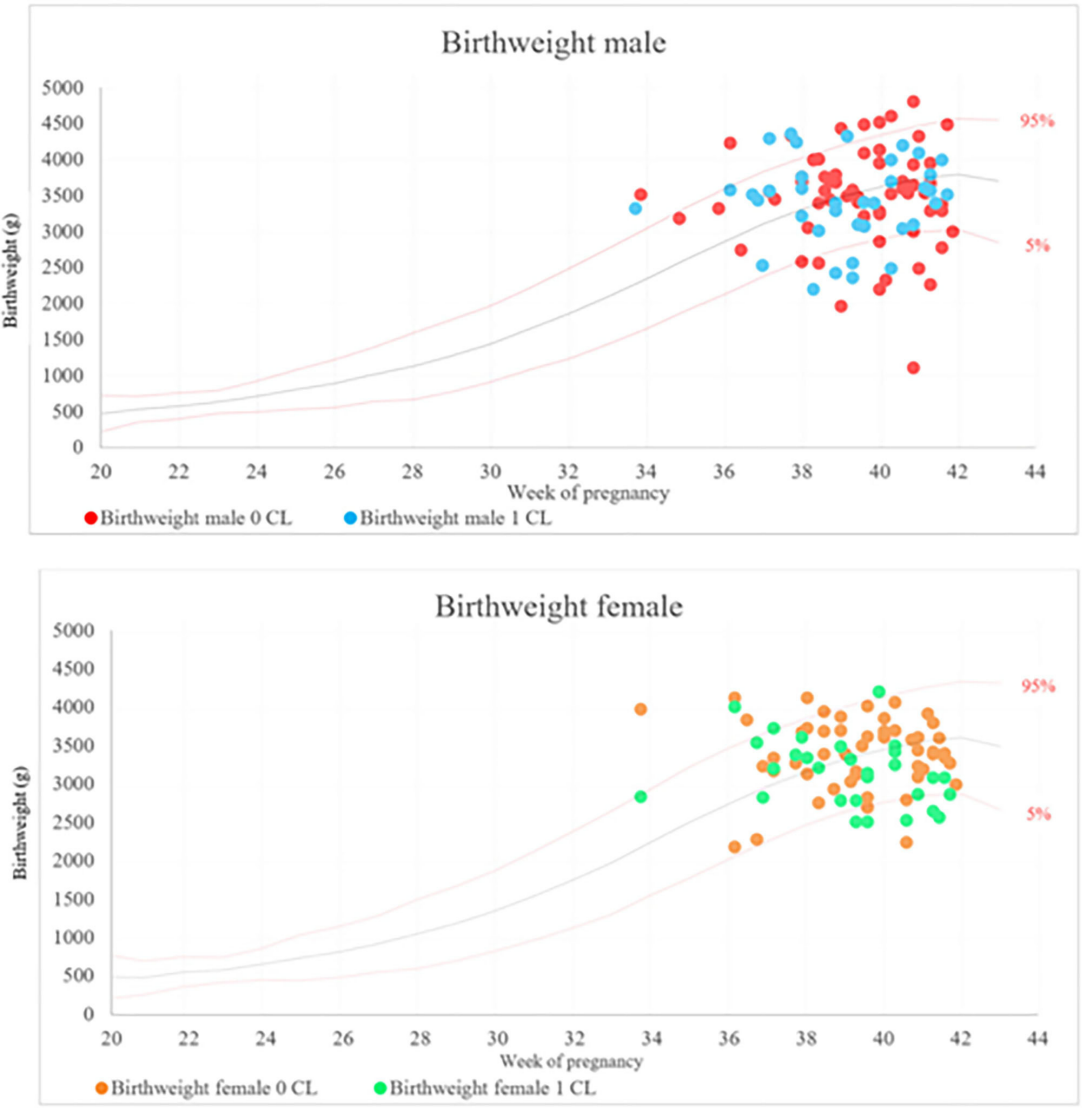
Birthweight of male and female newborn plotted against the gestational week for frozen-thawed embryo transfer in the absence (0 CL) or presence (≥1 CL) of a corpus luteum in German normality curves.

There were no differences for neonatal outcomes with regards to preterm birth, low and very low birthweight, macrosomia, SGA birthweight, LGA birthweight, neonatal morbidity, admission to ICU or NICU and major birth defects between the two cohorts (Table 4). Newborns conceived in the absence of a CL were on average 183 g heavier compared to conceptions in the presence of a CL (3,510 g ± 608 g vs. 3,327.5 g ± 735 g; *p* = 0.03). The same applies to the birthweight percentile with a mean difference of 9, presenting larger values for children born in the 0 CL cohort in comparison to children born in the ≥1 CL group (51.5 ± 41% vs. 42.5 ± 42%, *p* = 0.02) (Table 4). After adjustment for potential confounders, CL absence was a significant predictor of higher birthweight (adjusted coefficient B 179.74, 95% CI 13.03 – 346.44) and higher birthweight percentiles (adjusted coefficient B 10.23, 95% CI, 2.28 – 18.40) (Table 5). Figure 1 shows the birthweight values plotted on reference curves for the birthweight of a healthy German population with adjustment for gestational week and gender (31). In a sub-analysis birthweight and birthweight percentiles were higher in female but not male newborn of the 0 CL cohort than in the ≥1 CL cohort (*p* = 0.02) (Table 4). Female gender was a significant predictor of higher birthweight (adjusted coefficient B 267.35, 95% CI 53.64 – 481.07) and higher birthweight percentiles (adjusted coefficient B 17.85, 95% CI 5.90 – 29.80) in the 0 CL cohort compared to the ≥1 CL cohort (Table 6).

**Table 4:** Neonatal outcomes in singleton birth after frozen-thawed embryo transfer in the absence (0 CL) or presence (≥1 CL) of a corpus luteum^a^.

**Outcomes**	**0 CL**	**≥1 CL**	***P*-value**	**Crude OR (95% CI)**	***P*-value**	**Adjusted OR^**g**^**	***P*-value**
	**(*n* = 114)**	**(*n* = 68)**				**(95% CI)**	
Gestational age, days	277.0 ± 19^b^	276.5 ± 16	0.63	NA	NA	NA	NA
Gender				NA	NA	NA	NA
- Male	61 (53.5)^b^	38 (55.9)^d^	0.72				
- Female	52 (45.6)	29 (42.6)					
Preterm birth (<37 weeks)	14 (12.3)	7 (10.3)	0.67	1.23 (0.47 – 3.22)	0.67	1.07 (0.38 – 3.04)	0.90
Very preterm birth (<32 weeks)	1 (0.9)	0 (0.0)	1.0	NA	NA	NA	NA
Birthweight, g	3,510.0 ± 608^b^	3327.5 ± 735	0.03	NA	NA	NA	NA
Birthweight percentile (<1 - >99)	51.5 ± 41^e^	42.5 ± 42	0.02	NA	NA	NA	NA
Birthweight, g (male)	3,532.5 ± 69.8	3,430.0 +/- 685.0	0.45	NA	NA	NA	NA
Birthweight percentile (<1 - >99) (male)	47.5 ± 40.0	47.5 ± 45.0	0.58	NA	NA	NA	NA
Birthweight, g (female)	3402.3 ± 465.4^#^	3154.0 ± 440.9^#^	0.02	NA	NA	NA	NA
Birthweight percentile (<1 - >99) (female)	57.4 ± 40	37.0 ± 32	0.007	NA	NA	NA	NA
**Birthweight category**							
Low birthweight (<2,500 g)	9 (7.9)	4 (5.9)	0.77	1.39 (0.41 – 4.68)	0.60	1.28 (0.33 – 4.91)	0.72
Very low birthweight (<1,500 g)	2 (1.8)	0 (0.0)	0.53	NA	NA	NA	NA
Macrosomia (>4,000 g)	17 (14.9)	8 (11.8)	0.54	1.33 (0.54 – 3.27)	0.54	1.42 (0.55 – 3.70)	0.47
SGA (<10 percentile)	8 (7.0)	10 (14.7)	0.10	0.44 (0.17 – 1.18)	0.10	0.41 (0.14 – 1.23)	0.11
LGA (>90 percentile)	11 (9.6)	7 (10.3)	0.92	0.95 (0.35 – 2.58)	0.92	1.00 (0.36 – 2.77)	>0.9
Body size newborn - cm	52 ± 3^c^	51 ± 4^d^	0.06	NA	NA	NA	NA
Head circumference - cm	35.5 ± 2^c^	35.0 ± 2^d^	0.07	NA	NA	NA	NA
APGAR <7 at 5 min	2 (1.8)	0 (0.0)	0.53	NA	NA	NA	NA
Neonatal morbidity^f^	8 (7.0)	8 (11.8)	0.28	0.57 (0.20 – 1.60)	0.29	0.19 (0.16 – 1.44)	0.19
Admission to ICU or NICU	16 (14.0)	16 (23.5)	0.12	0.54 (0.25 – 1.17)	0.12	0.49 (0.22 – 1.09)	0.08
Major birth defects	4 (3.5)	3 (4.4)	0.75	0.78 (0.17 – 3.61)	0.75	0.63 (0.13 – 3.15)	0.59

a *Data are presented as median ± interquartile range or as number (% of total)*.

b *113/114 outcomes available*.

c *111/114 outcomes available*.

d *67/68 outcomes available*.

e *112/114 outcomes available*.

f *Icterus, infection, sepsis, infant respiratory distress syndrome, hypoglycemia*.

g *Adjustments were made for the following confounders: child's sex, nulliparity, HDP, placenta-associated complications, smoking during pregnancy, gestational diabetes*.

*CI, confidence interval; CL, corpus luteum; ICU, intermediate care unit; LGA, large for gestational age; NICU, intensive care unit; OR, odds ratio; SGA, small for gestational age*.

# *Means that this value is a mean +/− standard devation instead of a median value*.

**Table 5:** Neonatal outcome (crude and adjusted coefficient B) for singleton birth after frozen-thawed embryo transfer in the absence (*n* = 114) or presence (*n* = 68) of a corpus luteum.

**Outcome**	**Crude coefficient B (95% CI)**	***P*-value**	**Adjusted coefficient B^**a**^ (95% CI)**	***P*-value**
Birthweight, g	151.53 (-22.81 – 325.87)	0.09	179.74 (13.03 – 346.44)	0.04
Birthweight percentile (1–100)	9.87 (1.67 – 18.07)	0.02	10.23 (2.28 – 18.40)	0.01

a *Adjustments were made for the following confounders: child's sex, nulliparity, HDP, placenta-associated complications, smoking during pregnancy, gestational diabetes*.

*CI, confidence interval*.

**Table 6:** Birthweight and birthweight percentiles (crude and adjusted coefficient B) for singleton male and female birth after frozen-thawed embryo transfer in the absence (*n* = 114) or presence (*n* = 68) of a corpus luteum.

**Outcome**	**Crude coefficient B (95% CI)**	***P*-value**	**Adjusted coefficient B^**a**^ (95% CI)**	***P*-value**
Birthweight, g (male)	66.94 (-200.48 – 334.35)	0.2	133.86 (-125.09–329.81)	0.31
Birthweight percentile(<1 - >99) (male)	381.26 (-7.41 – 15.03)	0.67	6.16 (-5.39 – 17.71)	0.29
Birthweight, g (female)	248.27 (37.53 – 459.02)	0.02	267.35 (53.64 – 481.07)	0.02
Birthweight percentile(<1 - > 99) (female)	16.26 (4.08 – 28.44)	0.01	17.85 (5.90 – 29.80)	0.004

a *Adjustments were made for the following confounders: nulliparity, HDP, placenta-associated complications, smoking during pregnancy, gestational diabetes*.

*CI, confidence interval*.

### Comparison Between Survey Answers and Verified Diagnoses

The survey included questions on complications during the index pregnancy, e.g., the occurrence of hypertension, preeclampsia, HELLP-syndrome, gestational diabetes, or preterm birth. According to the questionnaire, 15 participants (8.2%) were diagnosed with hypertension during pregnancy, seven participants (3.8%) with preeclampsia, five participants (2.7%) with HELLP-syndrome, 19 participants (10.4%) participants with gestational diabetes, 24 participants (13.2%) with preterm birth, and 123 participants (67.6%) with none of the five diagnoses. The biggest difference in prevalence of survey answer and verified diagnosis was observed for gestational diabetes (1.7%) and for non-occurrence of the five diagnoses (−1.6%). Differences between survey answers and verified diagnoses were minimal for hypertension (1.1%), preeclampsia (−0.6%), HELLP-syndrome (0.5%), and preterm birth (1.1%).

When comparing survey answers and verified diagnoses, almost perfect agreement (Cohen's Kappa) was observed for preeclampsia (0.93), HELLP-syndrome (0.87), gestational diabetes (0.92), and preterm birth (0.87). Agreement was fair in terms of hypertension (0.38) and substantial in terms of non-occurrence of the five diagnoses (0.80). Ten participants (16.9%) reported not having been diagnosed with one of the five complications, albeit we detected at least one of the diagnoses in these participants' medical records. For further details, see Table 7.

**Table 7:** Comparison of complications between survey answers and verified diagnoses for singleton birth after frozen-thawed embryo transfer.

**Complication during index pregnancy**	**Prevalence survey answer, *n* (%)**	**Prevalence verified diagnosis, *n* (%)**	**Difference in prevalence**	**Agreement (Cohen's Kappa)**
Hypertension	15 (8.2)	13 (7.1)	1.1	0.38
Preeclampsia	7 (3.8)	8 (4.4)	−0.6	0.93
HELLP-syndrome	5 (2.7)	4 (2.2)	0.5	0.87
Gestational diabetes	19 (10.4)	22 (12.1)	−1.7	0.92
Preterm birth	24 (13.2)	22 (12.1)	1.1	0.87
Non-occurrence	123 (67.6)	127 (69.8)	−1.6	0.80

## Discussion

Our study evaluated the effect of CL absence in FET cycles on obstetric and neonatal outcomes in a German cohort, which included singleton deliveries. We confirmed a higher risk to develop HDP after FET lacking a CL compared to FET with CL presence. Additionally, the birthweight and birthweight percentiles were higher for children born after conceptions without a CL compared to children born after conceptions developing a CL. CL absence was a predictor of higher birthweight and birthweight percentiles particularly in female newborn.

A Cochrane review published in 2017 concluded that there was no clear evidence to suggest that one FET protocol is superior to another with respect to clinical efficacy outcomes (32). Hence, the decision which protocol to choose is often mainly on physician's preference. Due to the flexible planning, a large number of FET cycles is currently being performed in a programmed cycle regimen.

However, more recent studies suggest better outcomes with regards to clinical (19, 33–37), ongoing (19, 33, 38–40), and live birth (18, 19, 33, 34, 36–38, 40–43) rates in (modified) natural or stimulated FET cycles compared to cycles performed in an artificial setting. Nevertheless, these data are from different populations and quality and mainly from retrospective observational studies which prompts caution when drawing conclusions for clinical practice.

Vasoactive products of the CL, which are present in (modified) natural and stimulated FET cycles, have great relevance in maternal adaptive circulatory processes during early pregnancy. Recent findings indicate that a lack of relaxin-2—as described in patients lacking CL development (44)—results in attenuated increase in central arterial compliance and higher preeclampsia risk (3). Our findings support these data, although our primary outcome also includes HELLP-syndrome and gestational hypertension in addition to preeclampsia as one outcome summarized, so that these models are not fully comparable. However, according to our hypothesis, the incidence of HDP entities overall would be affected by CL absence, as a consequence of similar pathophysiology—demonstrated by the fact that preeclampsia is commonly defined as the new onset or worsening of hypertension during pregnancy (45), implying that gestational hypertension often acts as a precondition. Hence, up to 35% of patients with gestational hypertension develop preeclampsia (46). Our findings of an increased risk to develop HDP are in line with a growing body of evidence accumulating from mainly observational studies examining the impact of different FET protocols on maternal and neonatal outcomes (15, 16, 20, 47–51).

Previous studies report on the more frequent occurrence of LGA infants and macrosomia after programmed FET cycles (16, 43, 48) while we found higher birthweight and birthweight percentile after FET with CL absence as well as CL absence as a significant predictor of higher birthweight and birthweight percentiles.

A possible explanation for our finding is a lower estradiol level during FET preparation, compared to those in cycles involving CL presence. Especially in case of stimulated FET cycles, the ovarian stimulation might result in supraphysiologial estradiol levels (52). Several studies suggest that there is an association between this specific maternal hormonal environment and lower birthweights in comparison to programmed FET cycles (52–55). Additionally, the CL displays the main source of estrogen in early pregnancy until the placenta begins to contribute the hormone from about gestational week nine on (56), demonstrating another reason for lower estradiol levels in FET's without CL. Having detected differences not only in birthweights but in birthweight percentiles as well with the same tendency toward higher values in the 0 CL cohort implies that gestational age at birth does not play a role in this outcome but other factors must determine the differences. In our study female gender was a predictor of higher birthweight and birthweight percentile in the 0 CL cohort which hasn't been described before. In recent years several studies suggest that biological differences between the sexes are apparent already in early pregnancy including different placental gene, protein and micro RNA expression in adverse conditions (57). Other reports draw attention to further parameters that could have an impact on birthweight in ART pregnancies, such as *in vitro* culture (58), single embryo transfer (59), paternal BMI (60), and endometrial thickness (61), from which some of these factors were not included in our study. However, O'Neill et al. reported that the well-known sex-related differential growth after spontaneous conceptions is apparent but not exaggerated in conceptions from IVF treatments (62). Therefore, our observation of a sex-dependent difference in birth weight is of particular interest and the hypothesis that the lack of CL products might contribute to higher birthweights in female offspring warrants further exploration.

Our study could not demonstrate a significant difference in the incidences of postpartum hemorrhage and placenta-associated complications between both groups, likely due to small sample sizes. However, studies supporting a link between these outcomes and CL absence have recently accumulated (15, 16, 43). Since placenta accreta as well as similar placenta abnormalities mostly result from failure of decidualization, abnormal placentation and vascularisation as well as tissue oxygenation (63), CL absence could have a similar pathophysiological sequence, only with a different primary triggering event, which would be the absence of crucial CL products in this scenario. Progesterone regulates predecidualization and decidualization, enabling the endometrium to convert into a strongly vascularized form which, again, is the requirement for a physiological implantation and placentation process (64). Estrogen, relaxin and vascular endothelial growth factor among other CL products, contribute to these well-functioning processes, too (21, 23, 27, 65). It is therefore conceivable that CL absence could contribute to higher rates of placenta-associated complications. Abnormalities like placenta accreta usually correspond with higher postpartum hemorrhage rates (66), which are also more frequent after conceptions with CL absence, as mentioned above.

### Strengths and Limitations

Our study has a variety of limitations, including relatively small sample size and differing cohort sizes. These factors make it difficult to reliably show statistical correlations, especially because several outcomes that we analyzed already occur rarely. The fact that we did not find significantly higher risks for certain outcomes between the two cohorts might be due to this circumstance, albeit there is nonetheless a discernable tendency toward higher incidences for several adverse outcomes in the 0 CL group. Low sample size is also reflected in partly wide confidence intervals.

Another limiting parameter displays the retrospectivity of the study with its inherent recall bias, forcing us to rely on correctness of pre-existing data. However, compared to other observational and registry studies which relied on non-verified patient information, we were able to include reliable information of important parameters extracted from medical records which enabled us to adjust for relevant confounders that have a well-known effect on the studied outcomes.

Frequently, a retrospective study design is associated with risk for selection bias, which in case of our study would be most consequential when it applies to the FET protocol. However, in two of the fertility clinics involved mainly one FET protocol was in use (e.g., 93.5% of the participants treated in center 2 received a programmed cycle protocol, 97.7% at center 1 were treated in stimulated cycles), minimizing the risk of selection bias that occurs when for example PCOS patients very commonly receive a FET in programmed cycles. In our study, PCOS/anovulation diagnosis was represented equally in both cohorts. Nevertheless, certain infertility diagnoses like male factor infertility or recurrent pregnancy loss showed a significant difference in their distribution, which might be caused by the fertility clinics' divergent diagnostic procedure standards. Also, we focused our data collection on the responders and didn't analyze the characteristics of the non-responders. Therefore, we can't fully assure that the responders are a representative sample of patients and subtle differences between these groups might exist.

With our study design, randomization of the participants was not possible, so consequently, we attempted to reduce the potential effect of confounders on outcome events by adjustments made in multivariable regression analyses.

One of our study's great strengths is the fact that each primary outcome HDP diagnosis was verified blinded by an experienced obstetrician. On this basis, values from blood tests, urine samples and blood pressure measurements were evaluated and categorized with respect to gestational weeks, resulting in a precise reliable classification of HDP. During this process, cohort allocation was blinded, reducing the risk of detection bias. Verification of the cycle protocol followed the same pattern with assessment of each participant's drug administration.

Also, participants' response rates varied between 82.1% from participants previously treated at the academic center to 60.3% in one of the two private fertility centers. These rates could have been biased, possibly caused by different satisfaction rates in terms of the fertility treatment or by the fact that participants treated at two centers had a less personal connection to the staff of the fertility clinic which was in charge of the study and performed the recruitment than participants who had their fertility treatment at this facility.

Further questions touch upon potential associations between CL absence and higher rates of specific preeclampsia forms, e.g., early-onset preeclampsia (<34 gestational weeks) and preeclampsia with severe features. While one previous study suggests that CL absence was predictive of preeclampsia with severe features (3) this study does not allow definite answers about whether CL absence does have a significant impact on incidences of these subtypes and necessitates further research with larger sample sizes, too.

## Conclusion

We report a higher risk for HDP after FET in conceptions lacking a CL compared to FET in cycles involving CL presence. Additionally, CL absence was a predictor of higher birthweight and birthweight percentiles. Our findings support existing data suggesting a link between CL absence and adverse maternal outcomes, such as HDP. Maternal cardiovascular health and physiological adaptions during pregnancy are likely influenced by vasoactive products secreted by the CL. Further investigations comparing benefits and disadvantages of different FET cycle protocols are urgently needed in order to evaluate which treatment is associated with the best maternal and neonatal outcome, preferably using a randomized study design.

## Data Availability Statement

The raw data supporting the conclusions of this article will be made available by the authors, without undue reservation.

## Ethics Statement

The studies involving human participants were reviewed and approved by Ethics Committee (Institutional Review Board) of Hannover Medical School. The patients/participants provided their written informed consent to participate in this study.

## Author Contributions

FW, LK, and FV-H: acquisition and interpretation of data. FW, LK, and YZ: analysis. FW and FV-H: drafted the work for important intellectual content. CS, DT, AC, and TS: revised it critically for important intellectual content. All authors approved the version to be published, agreed to be accountable for all aspects of the work in ensuring that questions related to the accuracy or integrity of any part of the work are appropriately investigated and resolved, and made substantial contributions to the conception or design of the work.

## Funding

This study was supported by funds of Hannover Medical School, Department of Obstetrics and Gynecology.

## Conflict of Interest

The authors declare that the research was conducted in the absence of any commercial or financial relationships that could be construed as a potential conflict of interest.

## Publisher's Note

All claims expressed in this article are solely those of the authors and do not necessarily represent those of their affiliated organizations, or those of the publisher, the editors and the reviewers. Any product that may be evaluated in this article, or claim that may be made by its manufacturer, is not guaranteed or endorsed by the publisher.

## References

[1] De Geyter C Wyns C Calhaz-Jorge C de Mouzon J Ferraretti AP Kupka M. 20 years of the European IVF-monitoring Consortium registry: what have we learned? A comparison with registries from two other regions. Hum Reprod. (2020) 35:2832–49. 10.1093/humrep/deaa250 33188410 PMC7744162

[2] Lawrenz B Coughlan C Melado L Fatemi HM. The ART of frozen embryo transfer: back to nature! Gynecol Endrocrinol. (2020) 36:479–83. 10.1080/09513590.2020.1740918 32188299

[3] von Versen-Höynck F Schaub AM Chi Y-Y Chiu K-H Liu J Lingis M. Increased preeclampsia risk and reduced aortic compliance with *in vitro* fertilization cycles in the absence of a corpus luteum. Hypertension. (2019) 73:640–9. 10.1161/HYPERTENSIONAHA.118.12043 30636552 PMC6434532

[4] Ishihara O. Impact of frozen-thawed single-blastocyst transfer on maternal and neonatal outcome: an analysis of 277,042 single-embryo transfer cycles from 2008 to 2010 in Japan. Fertil Steril. (2014) 101:128–33. 10.1016/j.fertnstert.2013.09.025 24268706

[5] Luke B. Pregnancy and birth outcomes in couples with infertility with and without assisted reproductive technology: with an emphasis on US population-based studies. Am J Obstet Gynecol. (2017) 217:270–81. 10.1016/j.ajog.2017.03.012 28322775 PMC9761478

[6] Maheshwari A. Is frozen embryo transfer better for mothers and babies? Can cumulative meta-analysis provide a definitive answer? Hum Reprod Update. (2018) 24:35–58. 10.1093/humupd/dmx031 29155965

[7] Opdahl S. Risk of hypertensive disorders in pregnancies following assisted reproductive technology: a cohort study from the CoNARTaS group. Hum Reprod. (2015) 30:1724–31. 10.1093/humrep/dev090 25924655

[8] Sazonova A. Obstetric outcome in singletons after in vitro fertilization with cryopreserved/thawed embryos. Hum Reprod. (2012) 27:1343–50. 10.1093/humrep/des036 22362926

[9] Sha T. Pregnancy-related complications and perinatal outcomes resulting from transfer of cryopreserved versus fresh embryos *in vitro* fertilization: a meta-analysis. Fertil Steril. (2018) 109:330–42.e9. 10.1016/j.fertnstert.2017.10.019 29331236

[10] Sites CK Wilson D Barsky M Bernson D Bernstein IM Boulet S. Embryo cryopreservation and preeclampsia risk. Fertil Steril. (2017) 108:784–90. 10.1016/j.fertnstert.2017.08.035 28974308 PMC10999961

[11] Wertheimer A Hochberg A Krispin E Sapir O Ben-Haroush A Altman E. Frozen-thawed embryo transfer is an independent risk factor for third stage of labor complications. Arch Gynecol Obstet. (2021) 304:531–7. 10.1007/s00404-020-05935-2 33398506

[12] Berntsen S Pinborg A. Large for gestational age and macrosomia in singletons born after frozen/thawed embryo transfer (FET) in assisted reproductive technology (ART). Birth Defects Res. (2018) 110:630–43. 10.1002/bdr2.1219 29714057

[13] Terho AM Pelkonen S Opdahl S Romundstad LB Bergh C Wennerholm UB. High birth weight and large-for-gestational-age in singletons born after frozen compared to fresh embryo transfer, by gestational week: a Nordic register study from the CoNARTaS group. Hum Reprod. (2021) 36:1083–92. 10.1093/humrep/deaa304 33416878

[14] Wennerholm U-B Henningsen A-KA Romundstad LB Bergh C Pinborg A Skjaerven R. Perinatal outcomes of children born after frozen-thawed embryo transfer: a Nordic cohort study from the CoNARTaS group. Hum Reprod. (2013) 28:2545–53. 10.1093/humrep/det272 23832793

[15] Asserhøj LL Spangmose AL Aaris Henningsen A-K Clausen TD Ziebe S Jensen RB. Adverse obstetric and perinatal outcomes in 1,136 singleton pregnancies conceived after programmed frozen embryo transfer (FET) compared with natural cycle FET. Fertil Steril. (2021) 115:947–56. 10.1016/j.fertnstert.2020.10.039 33461756

[16] Ginström Ernstad E Wennerholm U-B Khatibi A Petzold M Bergh C. Neonatal and maternal outcome after frozen embryo transfer: Increased risks in programmed cycles. Am J Obstet Gynecol. (2019) 221:126.e1–18. 10.1016/j.ajog.2019.03.010 30910545

[17] Wang Z Liu H Song H Li X Jiang J Sheng Y. Increased risk of pre-eclampsia after frozen-thawed embryo transfer in programming cycles. Front Med-Lausanne. (2020) 7:104. 10.3389/fmed.2020.00104 32322584 PMC7156607

[18] Jing S Li XF Zhang S Gong F Lu G Lin G. Increased pregnancy complications following frozen-thawed embryo transfer during an artificial cycle. J Assist Reprod Gen. (2019) 36:925–33. 10.1007/s10815-019-01420-1 30924053 PMC6541721

[19] Lin J Zhao J Hao G Tan J Pan Y Wang Z. Maternal and neonatal complications after natural vs. hormone replacement therapy cycle regimen for frozen single blastocyst transfer. Front Med-Lausanne. (2020) 7:338. 10.3389/fmed.2020.00338 32984357 PMC7483478

[20] Makhijani R Bartels C Godiwala P Bartolucci A Nulsen J Grow D. Maternal and perinatal outcomes in programmed versus natural vitrified-warmed blastocyst transfer cycles. Reprod Biomed Online. (2020) 41:300–8. 10.1016/j.rbmo.2020.03.009 32505542

[21] Singh B Reschke L Segars J Baker VL. Frozen-thawed embryo transfer: the potential importance of the corpus luteum in preventing obstetrical complications. Fertil Steril. (2020) 113:252–7. 10.1016/j.fertnstert.2019.12.007 32106972 PMC7380557

[22] Wiegel RE Jan Danser AH Steegers-Theunissen RPM Laven JSE Willemsen SP Baker VL. Determinants of maternal renin-angiotensin-aldosterone-system activation in early pregnancy: insights from 2 cohorts. J Clin Endocr Metab. (2020) 105:3505–17. 10.1210/clinem/dgaa582 32853347 PMC7494245

[23] Conrad KP. Evidence for corpus luteal and endometrial origins of adverse pregnancy outcomes in women conceiving with or without assisted reproduction. Obstet Gyn Clin N Am. (2020) 47:163–81. 10.1016/j.ogc.2019.10.011 32008666 PMC7106658

[24] Conrad KP Petersen JW Chi Y-Y Zhai X Li M Chiu K-H. Maternal cardiovascular dysregulation during early pregnancy after *in vitro* fertilization cycles in the absence of a corpus luteum. Hypertension. (2019) 74:705–15. 10.1161/HYPERTENSIONAHA.119.13015 31352818 PMC6687559

[25] Dall'Agnol H García Velasco JA. Frozen embryo transfer and preeclampsia: where is the link? Curr Opin Obstet Gyn. (2020) 32:213–8. 10.1097/GCO.0000000000000624 32324715

[26] von Versen-Höynck F Häckl S Selamet Tierney ES Conrad KP Baker VL Winn VD. Maternal vascular health in pregnancy and postpartum after assisted reproduction. Hypertension. (2020) 75:549–60. 10.1161/HYPERTENSIONAHA.119.13779 31838910 PMC7491550

[27] Marshall SA Senadheera SN Parry LJ Girling JE. The role of relaxin in normal and abnormal uterine function during the menstrual cycle and early pregnancy. Reprod Sci. (2017) 24:342–54. 10.1177/1933719116657189 27365367

[28] von Versen-Höynck F Narasimhan P Selamet Tierney ES Martinez N Conrad KP Baker VL. Absent or excessive corpus luteum number is associated with altered maternal vascular health in early pregnancy. Hypertension. (2019) 73:680–90. 10.1161/HYPERTENSIONAHA.118.12046 30636549 PMC6378337

[29] AWMF-Leitlinie 015 / 018 (S2k). Diagnostik und Therapie hypertensiver Schwangerschaftserkrankungen. AWMF (2019). Available online at: https://www.awmf.org/uploads/tx_szleitlinien/015-018l_S2k_Diagnostik_Therapie_hypertensiver_Schwangerschaftserkrankungen_2019-07.pdf (accessed March 28, 2021).

[30] Gestational hypertension and preeclampsia: ACOG practice bulletin number 222. Obstet Gynecol. (2020) 135:e237–60. 10.1097/AOG.0000000000003891 32443079

[31] Voigt M. Methodische Aspekte der Berechnung von Normwertkurven für das Geburtsgewicht. Geburtshilfe Frauenheilkd. (2005) 65:279–83. 10.1055/s-2005-837528

[32] Ghobara T Gelbaya TA Ayeleke RO. Cycle regimens for frozen-thawed embryo transfer. Cochrane DB Syst Rev. (2017) 7:CD003414. 10.1002/14651858.CD003414.pub3 28675921 PMC6483463

[33] Guan Y Fan H Styer AK Xiao Z Li Z Zhang J. modified natural cycle results in higher live birth rate in vitrified-thawed embryo transfer for women with regular menstruation. Syst Biol Reprod Med. (2016) 62:335–42. 10.1080/19396368.2016.1199064 27400398

[34] Huang P Wei L Li X Lin Z. Modified hMG stimulated: an effective option in endometrial preparation for frozen-thawed embryo transfer in patients with normal menstrual cycles. Gynecol Endocrinol. (2018) 34:772–4. 10.1080/09513590.2018.1460342 29676585

[35] Orvieto R Feldman N Lantsberg D Manela D Zilberberg E Haas J. Natural cycle frozen-thawed embryo transfer-can we improve cycle outcome? J Assist Reprod Gen. (2016) 33:611–5. 10.1007/s10815-016-0685-5 26973337 PMC4870445

[36] Tatsumi T Jwa SC Kuwahara A Irahara M Kubota T Saito H. Pregnancy and neonatal outcomes following letrozole use in frozen-thawed single embryo transfer cycles. Hum Reprod. (2017) 32:1244–8. 10.1093/humrep/dex066 28398491

[37] Wang B Zhu Q Wang Y. Pregnancy outcomes after different cycle regimens for frozen-thawed embryo transfer: a retrospective study using propensity score matching. Front Med-Lausanne. (2020) 7:327. 10.3389/fmed.2020.00327 32850875 PMC7399073

[38] Peigné M Devouche E Ferraretto X Gricourt S Luton D Patrat C. Higher live birth rate with stimulated rather than artificial cycle for frozen-thawed embryo transfer. Eur J Obstet Gyn R B. (2019) 243:144–9. 10.1016/j.ejogrb.2019.10.040 31704531

[39] Wang A Murugappan G Kort J Westphal L. Hormone replacement versus natural frozen embryo transfer for euploid embryos. Arch Gynecol Obstet. (2019) 300:1053–60. 10.1007/s00404-019-05251-4 31338657

[40] Zhang J Liu H Wang Y Mao X Chen Q Fan Y. Letrozole use during frozen embryo transfer cycles in women with polycystic ovary syndrome. Fertil Steril. (2019) 112:371–7. 10.1016/j.fertnstert.2019.04.014 31126712

[41] Liu X Shi W Shi J. Natural cycle frozen-thawed embryo transfer in young women with regular menstrual cycles increases the live-birth rates compared with hormone replacement treatment: a retrospective cohort study. Fertil Steril. (2020) 113:811–7. 10.1016/j.fertnstert.2019.11.023 32147171

[42] Melnick AP Setton R Stone LD Pereira N Xu K Rosenwaks Z. Replacing single frozen-thawed euploid embryos in a natural cycle in ovulatory women may increase live birth rates compared to medicated cycles in anovulatory women. J Assist Reprod Gen. (2017) 34:1325–31. 10.1007/s10815-017-0983-6 28647784 PMC5633571

[43] Saito K Kuwahara A Ishikawa T Morisaki N Miyado M Miyado K. Endometrial preparation methods for frozen-thawed embryo transfer are associated with altered risks of hypertensive disorders of pregnancy, placenta accreta, and gestational diabetes mellitus. Hum Reprod. (2019) 34:1567–75. 10.1093/humrep/dez079 31299081

[44] von Versen-Höynck F Strauch NK Liu J Chi Y-Y Keller-Woods M Conrad KP. Effect of mode of conception on maternal serum relaxin, creatinine, and sodium concentrations in an infertile population. Reprod Sci. (2019) 26:412–9. 10.1177/1933719118776792 29862889 PMC6728556

[45] Hariharan N Shoemaker A Wagner S. Pathophysiology of hypertension in preeclampsia. Microvasc Res. (2017) 109:34–7. 10.1016/j.mvr.2016.10.002 27793558

[46] Magee LA von Dadelszen P. State-of-the-art diagnosis and treatment of hypertension in pregnancy. Mayo Clin Proc. (2018) 93:1664–77. 10.1016/j.mayocp.2018.04.033 30392546

[47] Hu K-L Zhang D Li R. Endometrium preparation and perinatal outcomes in women undergoing single-blastocyst transfer in frozen cycles. Fertil Steril. (2021) 115:1487–94. 10.1016/j.fertnstert.2020.12.016 33487443

[48] Wang B Zhang J Zhu Q Yang X Wang Y. Effects of different cycle regimens for frozen embryo transfer on perinatal outcomes of singletons. Hum Reprod. (2020) 35:1612–22. 10.1093/humrep/deaa093 32681726

[49] Zaat TR Brink AJ de Bruin J-P Goddijn M Broekmans FJM Cohlen BJ. Increased obstetric and neonatal risks in artificial cycles for frozen embryo transfers? Reprod Biomed Online. (2021) 42:919–29. 10.1016/j.rbmo.2021.01.015 33736993

[50] Zhang J Wei M Bian X Wu L Zhang S Mao X. Letrozole-induced frozen embryo transfer cycles are associated with a lower risk of hypertensive disorders of pregnancy among women with polycystic ovary syndrome. Am J Obstet Gynecol. (2021) 225:59.e1–9. 10.1016/j.ajog.2021.01.024 33529574

[51] Zong L Liu P Zhou L Wei D Ding L Qin Y. Increased risk of maternal and neonatal complications in hormone replacement therapy cycles in frozen embryo transfer. Reprod Biol Endocrin. (2020) 18:36. 10.1186/s12958-020-00601-3 32366332 PMC7199365

[52] Cai J Liu L Xu Y Liu Z Jiang X Li P. Supraphysiological estradiol level in ovarian stimulation cycles affects the birthweight of neonates conceived through subsequent frozen-thawed cycles: a retrospective study. BJOG-Int J Obstet GY. (2019) 126:711–8. 10.1111/1471-0528.15606 30628169

[53] Luo L Jie H Chen M Zhang L Xu Y. Further evidence that a supraphysiologic estradiol level during ovarian stimulation affects birthweight: findings of fresh and frozen embryo transfer with comparable estradiol levels on human chorionic gonadotropin trigger. Gynecol Endocrinol. (2020) 37:422–7. 10.1080/09513590.2020.1811963 32865049

[54] Tarlatzi T Venetis C Sassi A Devreker F Englert Y Delbaere A. Higher estradiol levels are associated with lower neonatal birthweight after fresh and frozen embryo transfers. A cohort study of 3631 singleton IVF pregnancies. Gynecol Endocrinol. (2021) 37:618-623. 10.1080/09513590.2020.1827383 33016794

[55] Zhang W Ma Y Xiong Y Xiao X Chen S Wang X. Supraphysiological serum oestradiol negatively affects birthweight in cryopreserved embryo transfers: a retrospective cohort study. Reprod Biomed Online. (2019) 39:312–20. 10.1016/j.rbmo.2019.04.015 31255605

[56] Berkane N Liere P Oudinet J-P Hertig A Lefèvre G Pluchino N. From pregnancy to preeclampsia: a key role for estrogens. Endocr Rev. (2017) 38:123–44. 10.1210/er.2016-1065 28323944

[57] Alur PP. Sex differences in nutrition, growth, and metabolism in preterm infants. Front Pediatr. (2019) 7:22. 10.3389/fped.2019.00022 30792973 PMC6374621

[58] Dumoulin JC Land JA Van Montfoort AP Nelissen EC Coonen E Derhaag JG. Effect of *in vitro* culture of human embryos on birthweight of newborns. Hum Reprod. (2010) 25:605–12. 10.1093/humrep/dep456 20085915

[59] De Sutter P Delbaere I Gerris J Verstraelen H Goetgeluk S Van der Elst J. Birthweight of singletons after assisted reproduction is higher after single- than after double-embryo transfer. Hum Reprod. (2006) 21:2633–7. 10.1093/humrep/del247 16785258

[60] Ma M Zhang W Zhang J Liang Z Kuang Y Wang Y. Effect of paternal body mass index on neonatal outcomes of singletons after frozen-thawed embryo transfer cycles: analysis of 7,908 singleton newborns. Fertil Steril. (2020) 113:1215–23.e1. 10.1016/j.fertnstert.2020.02.100 32402450

[61] Zhang J Liu H Mao X Chen Q Si J Fan Y. Effect of endometrial thickness on birthweight in frozen embryo transfer cycles: an analysis including 6181 singleton newborns. Hum Reprod. (2019) 34:1707–15. 10.1093/humrep/dez103 31398256

[62] O'Neill KEKE. Sex-related growth differences are present but not enhanced in in vitro fertilization pregnancies. Fertil Steril. (2014) 101:407–12. 10.1016/j.fertnstert.2013.10.011 24220702 PMC3939682

[63] Jauniaux E Jurkovic D. Placenta accreta: pathogenesis of a 20th century iatrogenic uterine disease. Placenta. (2012) 33:244–51. 10.1016/j.placenta.2011.11.010 22284667

[64] Okada H Tsuzuki T Murata H. Decidualization of the human endometrium. Reprod Med Biol. (2018) 17:220–7. 10.1002/rmb2.12088 30013421 PMC6046526

[65] Young SL. Oestrogen and progesterone action on endometrium: a translational approach to understanding endometrial receptivity. Reprod Biomed Online. (2013) 27:497–505. 10.1016/j.rbmo.2013.06.010 23933037 PMC3818404

[66] Obstetric care consensus no. 7 summary: placenta accreta spectrum. Obstet Gynecol. (2018) 132:1519–21. 10.1097/AOG.0000000000002984 30461691

