# Supplementary material for: Pregnancy Outcomes After Frozen-Thawed Embryo Transfer in the Absence of a Corpus Luteum
Source: Front Med (Lausanne). 2021 Sep 10;8:727753. doi: 10.3389/fmed.2021.727753 (PMC8460906; doi:10.3389/fmed.2021.727753)
Supplement: Supplementary file 1 [file Data_Sheet_1.DOCX]

Supplementary Material

**Supplementary Figure 1.** Study flow diagram.
